# Identification of tetrodotoxin-producing *Shewanella* spp. from feces of food poisoning patients and food samples

**DOI:** 10.1186/1757-4749-5-15

**Published:** 2013-06-20

**Authors:** Duochun Wang, Yonglu Wang, Hongnan Huang, Jie Lin, Di Xiao, Biao Kan

**Affiliations:** 1 State Key Laboratory for Infectious Disease Prevention and Control/Collaborative Innovation Center for Diagnosis and Treatment of Infectious Diseases, National Institute for Communicable Disease Control and Prevention, Chinese Center for Disease Control and Prevention, P.O. Box 5, Beijing, Changping, China; 2 Ma’anshan Center for Disease Control and Prevention, Ma’anshan, China; 3 Center for Diseases Control and Prevention of Fujian Province, Fujian, China

**Keywords:** Tetrodotoxin, *Shewanella*, Food poisoning

## Abstract

*Shewanella* spp. is infrequently recovered from clinical specimens. Following two outbreaks of food poisoning, eight *Shewanella* spp. strains were obtained from the fecal specimens of patients, food and food processing-related materials. Tetrodotoxin (TTX) was identified in the culture supernatants of these strains, and the toxin’s biological activity was detected using a mouse bioassay. This study suggested that *Shewanella* strains can colonize and survive in human intestines. The study also raises the issues of the accumulation of TTX produced by *Shewanella* in food and the possible role of TTX-producing *Shewanella* in food poisoning.

## Background

*Shewanella* spp. is Gram-negative, motile bacilli that belong to the order Alteromonadales and the family Alteromonadceae, within the gamma subdivision of the Proteobacteria. Human infections with members of the genus *Shewanella* are rare and mainly involve the ears [1] and soft tissue [2-5]. The isolation of *Shewanella* spp. from patients with diarrhea, poultry and livestock has also been reported [6]. In most instances, the isolation of has occurred in the absence of clinical disease and has been considered to involve only colonization, rather than an active infection [7]. However, in recent years, it was found that *Shewanella* spp. can produce tetrodotoxin (TTX) [8]. TTX and its analogs (TTXs) have been detected in a wide variety of marine animals [9]. It has been suggested that TTX is a secondary metabolite produced by symbiotic bacteria in marine organisms that gradually accumulates in the bodies of marine organisms through the food chain [8,10]. With the source of TTX still a controversial issue, the exact origin of TTX in the food chain is unknown.

### Study design and results

**Figure 1:**
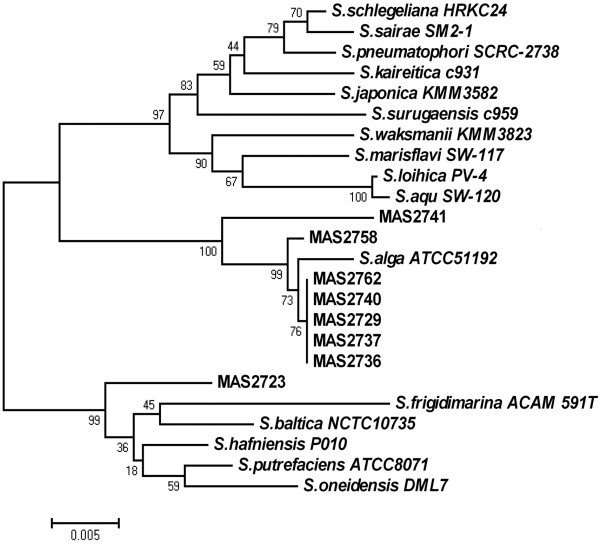
**16S rDNA gene phylogenetic tree of *Shewanella *spp. strains from this study and GenBank.** The GenBank accession numbers of the 16S rDNA of eight *Shewanella* spp. strains, MAS2723, MAS2729, MAS2736, MAS2737, MAS2740, MAS2741, MAS2758 and MAS2762, are GQ372872-79. Bar, 0.005 substitutions per nucleotide position.

On Sept. 29th and Oct. 2nd, 2007, two food poisoning incidents occurred following banquet dinners at two different restaurants in Ma’anshan City, China. A total of 50 people attended one dinner, and 152 people attended the other dinner. Of these individuals, 16 and 22 people, respectively, showed symptoms of food poisoning in 1–4 h, with abdominal pain (n=35), diarrhea (n=35, usually more than five times and consisting of yellow, watery stool), vomiting (n=24), nausea (n=16), poor peripheral circulation with dizziness (n=20) and/or headache (n=14) lasting for several hours. Specimens were collected in these two poisoning investigations and inoculated in selective media (WS, SS, EMB or TCBS Agar) after bacterial enrichment (see Additional file 1: Materials and methods). No common intestinal pathogens were isolated, such as *Vibrio cholerae*, *Salmonella*, *Shigella* and enteropathogenic *Escherichia coli*, but eight strains of *Shewanella* spp. were isolated from the specimens, which included hand swabs of a restaurant chef (strains MAS2723 and MAS2762), swabs of a restaurant knife (MAS2729), anal swabs of four patients (MAS2736, MAS2737, MAS2740 and MAS2741) and marinated beef (MAS2758). These strains were identified with biochemical tests and 16S rDNA sequencing (Figure 1: seven strains were *S. algae*, except strain MAS2723, which might be a new species of *Shewanella*).

**Figure 2:**
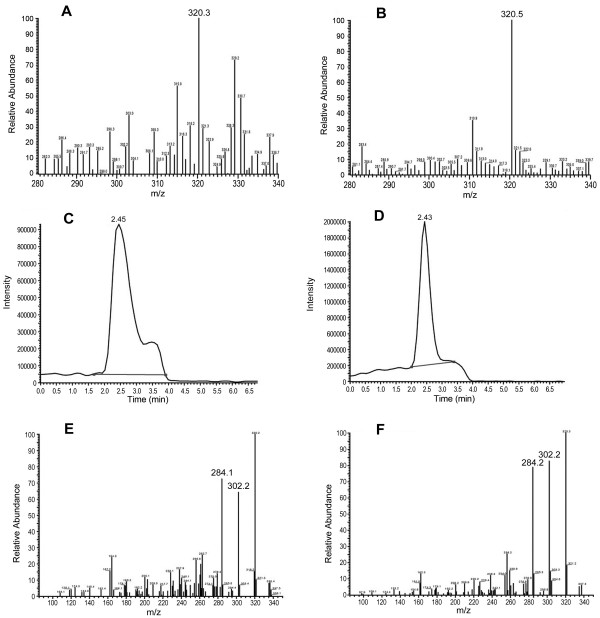
**Mass Spectrometry analysis of the TTX extracts from the *Shewanella *isolates. A** and **B** show the mass spectra of the sample containing TTX and of authentic TTX with ESI full MS. The retention time (RT) of the chromatogram of the sample, with a selective ion mode (SIM) specific for m/z 320 ions, was 2.45 min, which was similar to the RT (2.43 min) of authentic TTX (**C** and **D**). **E** and **F** show the MS/MS spectra of the sample containing TTX and of authentic TTX.

Because these patients were food poisoned and because TTX production has been reported for *Shewanella* strains [8], we measured TTX production by these isolates. TTX was detected from the seven-day culture supernatants by mass spectrometry assays (Figure 2). TTX extracts from cells and supernatants of the *Shewanella* spp. isolates were further subjected to an activity bioassay in mice (Table 1). After injection, TTX extracts from bacterial overnight cell cultures and supernatants and from seven-day cell cultures of all isolates did not induce symptoms of poisoning in the mice. In contrast, injection with TTX extracts from supernatants of seven-day cultures of all isolates caused the mice to show the same symptoms as those induced by a TTX standard in 0.5-13 minutes, including gait disorders, hindlimb weakness, convulsion and death. The mice in the control group had no obvious symptoms following the injection of 0.1% acetic acid.

**Table 1:** **Mouse bioassay of the toxicity of TTX extracts from the *Shewanella *spp. isolates**

**Item**	**Individual**	**Average**	**Mortality (%)**	**Mean lethal**
		**weight (g)**		**time (min)**
TTX extracts from cells	24	20.7	0	-
(overnight culture, 8 isolates)				
TTX extracts from supernatants	24	20.3	0	-
(overnight culture, 8 isolates)				
TTX extracts from cells (7-day	24	19.8	0	-
culture, 8 strains)				
TTX extracts from supernatants	24	20.6	100	2.5
(7-day culture, 8 isolates)				
TTX standard, 2.2 ***μ***g/ml	3	20.4	100	3
0.1% acetic acid	3	19.7	0	-

## Conclusion

The *Shewanella* strains recovered from the fecal samples of the food poisoning patients may present evidence of the survival of TTX-producing *Shewanella* in human intestines. To date, there is still no direct evidence to show that TTX produced by *Shewanella* spp. is related to food poisoning. Considering a previous report of *Shewanella* isolation from food poisoning patients [6] and our investigation, in which strains were also obtained from food, hand swabs of a restaurant chef and a knife, these findings suggest contamination with *Shewanella* through the fecal-oral route and indicate the need for detection and studies of *Shewanella* in food poisoning, especially if caused by TTX. The limitations of this study are that no blood samples were obtained for the detection of anti-TTX antibody, and no food sample was screened for TTX. Studies indicate that TTX accumulation in the puffer fish occurs through the food chain, consisting of several steps and starting with marine bacteria as the primary source [8]. In this study, very weak, slow TTX production by the *Shewanella* strains under common culture conditions was also observed. We suspected that weak accumulation of TTX in seafood might have caused the poisoning symptoms of the patients, as the recovered *Shewanella* strains were in fact the bacteria colonizing the seafood.

## Competing interests

The authors declare that they have no competing interests.

## Authors’ contributions

Conceived and designed the experiments: BK DW. Performed the experiments: DW YW HH JL DX. Analyzed the data: DW YW HH JL. Contributed reagents: HH JL. Discussion and wrote the paper: DW YW BK. All authors read and approved the final manuscript.

## Supplementary Material

Additional file 1 Materials and methods. Click here for file
