# Supplementary material for: Identification of tetrodotoxin-producing Shewanella spp. from feces of food poisoning patients and food samples
Source: Gut Pathog. 2013 Jun 20;5:15. doi: 10.1186/1757-4749-5-15 (PMC3734009; doi:10.1186/1757-4749-5-15)
Supplement: Additional file 1 — Materials and methods. [file 1757-4749-5-15-S1.docx]

**Materials and methods**

**Brief epidemiological data and samples collection**

On Sep. 29th and Oct. 2nd, 2007, two food poisoning incidents occurred from banquet dinners in two different restaurants of Ma’anshan City. The total of 50 and 152 people attended the two dinners respectively. Sixteen and 22 people showed the symptoms of food poisoning in 1-4 h and were treated in the emergency room of a local hospital. Twenty-nine and 34 specimens were collected in these two poisoning investigations respectively, including anal swabs and vomits from patients of nine and 10 specimens, respectively; Anal swabs, hand-swabs from chefs and tool swabs of nine and 10 specimens, respectively; Food and surplus food of five and 10 specimens, respectively.

**Bacterial isolation and biochemical identification**

Twenty-five grams of each food sample were cut or broken into 225 ml of isotonic NaCl and ground. Anal swabs of patients, the hotel chef hand painting and food knife tools painting were directly enriched without treatment. All samples were enriched in Miller-Mallinson (MM), Gram Negative, 7.5% Sodium Chloride and Sodium Chloride Violet purple Enrichment Broths, at 37°C (MM at 42°C) for 24 h before plating. About five μl of enriched broth were inoculated on WS Salmonella (WS), Salmonella Shigella (SS), Eosin Methylene Blue (EMB) and Thiosulfate Citrate Bile Salts Sucrose (TCBS) Agar, respectively, and incubated at 37°C for 18 to 24 h.

In order to isolate the possible pathogenic bacteria, colonies on the media were inoculated to triple sugar iron (TSI) agar, and incubated at 37°C for 18 to 24 h. Strains were identified by Gram staining and cytochrome oxidase activity. All strains were tested on VITEK-32 and the API 20E system (bioMe′rieux, France) for standard biochemical tests according to the manufacturer’s instructions.

**Amplification of 16S rRNA gene and sequencing**

Genomic DNA of bacterium was extracted using the DNA isolation kit (Qiagen DNA Mini Kit, Valencia, USA). 16S rRNA genes were amplified by PCR using the conserved primers (Weisburg et al., 1991) pF: AGAGTTTGATCCTGGCTCAG, and pR: ACGGCTACCTTGTTACGACTT. PCR was performed by initial denaturation at 94°C for 10 min followed by 30 cycles of 94°C for 30 sec, 58°C for 40 sec, and extension at 72°C for 1.5 min. A subsequent extension step at 72°C was run for 5 min.

QIAquick PCR columns (Qiagen) were used to purify the amplicons, which were approximately 1,510 bp. Then the amplicons were sequenced. Phylogenetic analysis of the 16S rRNA gene sequences of *Shewanella* spp. were carried out using the Lasergene software (DNASTAR). Multiple alignment of the nucleotide sequences was performed using Clustal-W. Gene trees showing percentage nucleotide substitution were generated with Mega 4 software [1].

**Purification of TTX from bacteria cultures**

Purification of TTX from bacteria cultures was performed according to Wu [2] and Yu [3] with some modification, briefly described as follow: Bacteria cultures were grown in 2216E medium (BD) in 500 ml volumes and incubated at 37°C. Both overnight and seven days cultures were collected; the broth cultures were centrifuged to obtain pellet and supernatant. The pellet was eluted with 50ml of 1% acetic acid and then broke the cell with ultra-wave generator; the mixture was heat with boiling water bath and then cooled at room temperature. The mixture was centrifugation to remove precipitate, the elution was freeze-drying and resuspended with 0.1% acetic acid; filtered with 3000 mol. Wt cut-off filter (YM30, 30000MWCO, Millipore) to collect of TTX.

The supernatant was evaporated using a rotary evaporator and eluted with 40 ml of 1% acetic acid in 20% aqueous ethanol, the elution was evaporated and lyophilized then elution with 15 ml of 0.1% acetic acid; elution was filtered same as pellet, to collect filtrate of TTX, which was subjected to further toxicity bioassay and detection of TTX by chemical analyses.

**High-performance liquid chromatography-mass spectrometry (HPLC-MS)**

LC-MS was carried out using a system equipped with a Surveyor LC system (Thermo Finnigan, San Jose, CA, USA). The LC separation was performed on a Thermo BioBasic-18 Pioneer (150mm×2.1mm, Particle size 5μm) column using 0.05mol/l acetic acid-methanol (95:5) as the mobile phase at a flow rate of 0.2ml/min. The column temperature was set at 38°C, and the injection volume was 10 μl. The TTX standard was dissolved with 0.05mol/l acetic acid, working concentration was 1μg/ml.

The eluate from the HPLC was passed through a PDA and then directly introduced into the mass spectrometer using electrospray ionization (ESI). All the analyses were performed using an ESI interface with the following setting: positive ionization mode; temperature of the capillary 275°C; spray voltage 4.5 kV; capillary voltage 28 V; sheath gas (N_2_) flows at 60 AU; aux gas (He) flows at 5AU. The ESI interface and mass spectrometric parameters were optimized to obtain maximum sensitive unit resolution. Analyst software is the Xcalibur 1.3 workstation, which was used for instrument control as well as data processing and analysis. The HPLC-MS analysis was accomplished with three of simultaneous acquisitions: (1) ions acquisition along time (TIC mode, Total Ion Current); (2) search for specific ions (SIM mode, Selective Ion Mode); (3) fragmentation using an inert gas.

**Mouse bioassay of TTX activity**

Mouse bioassay of TTX activity was carried according to Yu [3]. TTX standard (part number 630-002-M001, Axxora, Grünberg, Germany.) was eluated at the concentration of 2.2μg/ml. Injected intraperitoneally with male ICR strain mice weight around 20 g, the injected dose of one ml, symptoms were noted and the death time and weight were recorded at the last gasping breath of the mouse. Standard curve was drawn according to the countdown of death time-mice injected dose per body weight of TTX. Bacteria cells and culture supernatants extracts of TTX (both overnight and seven days) were injected mice in the same manner; the negative control is 0.1% acetic acid.

**Nucleotide sequence accession numbers**

GenBank accession numbers for the 16S rRNA gene sequences of strains were deposited in the database as follows: GQ372872 (strain MAS2723), GQ372873 (MAS2729), GQ372874 (MAS2736), GQ372875 (MAS2737), GQ372876 (MAS2740), GQ372877 (MAS2741), GQ372878 (MAS2758), and GQ372879 (MAS2762).

**Ethics statement**

This study was performed under the approval from the Ethics Committee of National Institute for Communicable Disease Control and Prevention, Chinese Center for Disease Control and Prevention. All animals were treated in strict according to the Rules for the Medical Laboratory Animal (1998) from Ministry of Health. In this study, when the experiment is finished, the mice still alive were killed by cervical dislocation after anesthesia. The Human fecal specimens were treated according to the Medical Research Regulations of Ministry of Health (permit number 2007-17-3). The anal swab samples of patients used in this study were obtained from the clinical laboratories when the patients were treated by the clinicians in the hospitals of after Ma’anshan city.

1. Tamura K, Dudley J, Nei M, Kumar S: **MEGA4: Molecular Evolutionary Genetics Analysis (MEGA) software version 4.0**. *Molecular biology and evolution* 2007, **24**(8):1596-1599.

2. Wu Z, Yang Y, Xie L, Xia G, Hu J, Wang S, Zhang R: **Toxicity and distribution of tetrodotoxin-producing bacteria in puffer fish Fugu rubripes collected from the Bohai Sea of China**. *Toxicon* 2005, **46**(4):471-476.

3. Yu CF, Yu PH, Chan PL, Yan Q, Wong PK: **Two novel species of tetrodotoxin-producing bacteria isolated from toxic marine puffer fishes**. *Toxicon* 2004, **44**(6):641-647.
